# Polyaniline cryogels: Biocompatibility of novel conducting macroporous material

**DOI:** 10.1038/s41598-017-18290-1

**Published:** 2018-01-09

**Authors:** Petr Humpolíček, Katarzyna Anna Radaszkiewicz, Zdenka Capáková, Jiří Pacherník, Patrycja Bober, Věra Kašpárková, Petra Rejmontová, Marián Lehocký, Petr Ponížil, Jaroslav Stejskal

**Affiliations:** 1 Centre of Polymer Systems, Tomas Bata University in Zlin, 760 01 Zlin, Czech Republic; 2 Faculty of Technology, Tomas Bata University in Zlin, 760 01 Zlin, Czech Republic; 3 Institute of Experimental Biology, Faculty of Science, Masaryk University, 625 00 Brno, Czech Republic; 4 Institute of Macromolecular Chemistry, Academy of Sciences of the Czech Republic, 162 06 Prague 6, Czech Republic

## Abstract

Polyaniline cryogel is a new unique form of polyaniline combining intrinsic electrical conductivity and the material properties of hydrogels. It is prepared by the polymerization of aniline in frozen poly(vinyl alcohol) solutions. The biocompatibility of macroporous polyaniline cryogel was demonstrated by testing its cytotoxicity on mouse embryonic fibroblasts and *via* the test of embryotoxicity based on the formation of beating foci within spontaneous differentiating embryonic stem cells. Good biocompatibility was related to low contents of low-molecular-weight impurities in polyaniline cryogel, which was confirmed by liquid chromatography. The adhesion and growth of embryonic stem cells, embryoid bodies, cardiomyocytes, and neural progenitors prove that polyaniline cryogel has the potential to be used as a carrier for cells in tissue engineering or bio-sensing. The surface energy as well as the elasticity and porosity of cryogel mimic tissue properties. Polyaniline cryogel can therefore be applied in bio-sensing or regenerative medicine in general, and mainly in the tissue engineering of electrically excitable tissues.

## Introduction

It is no exaggeration to say that the impact of electricity on living subjects has been known at least since 1792, when Luigi Galvani noticed that an accidental spark discharge caused frog muscle fibres to contract. This was one of the first studies related to bioelectricity. Since that day, the knowledge on bioelectricity, which studies electrical signals from excitable tissues, has increased continuously. Today it is well known that bioelectricity plays a crucial role in a variety of cellular processes, such as cell division, cell signalling, and differentiation, as well as on the tissue level, e.g., in wound healing or angiogenesis. Knowledge of bioelectricity has led to the application of conducting materials in regenerative medicine and the tissue engineering of electrically excitable tissues, as well as in the field of bio-sensing.

Considering applications in bio-sensing, conventional materials such as platinum, gold or iridium oxide ensure good mechanical properties in sensors, e.g., hardness and shape; however, their properties are highly dissimilar to those of native tissues. This can lead to irritation, and adverse immune responses of tissues in contact with such materials. Because of this, conducting polymers (CP), such as polyaniline, polypyrrole or poly(3,4-ethylenedioxythiophene), are considered as promising materials which can offer improved properties in comparison with conventional ones. The above-mentioned problems are very well described in the review by Jeong *et al*.^1^ Moreover, it is known that the charge-transfer characteristics of the conventional metal electrodes used in bio-sensing are improved when coated with conducting polymers, which exhibit combined electronic and ionic conductivity^2–4^.

The applicability of conducting polymers in tissue engineering is closely related to the preparation of corresponding 3D structures, ideally in a form combining appropriate biological as well as both mechanical and surface properties. As conducting polymers do not have the required bulk parameters, they have to be combined with other biopolymers or biomaterials^5–7^. Moreover, different routes for the preparation of 3D structures based on different types of CP can result in a broad variety of final materials which have different properties, this variability having a significant impact on biocompatibility and cell behaviour. Various preparation routes for CP-based 3D structures can involve, for example, conducting materials of a nanofibrous character prepared via electrospinning, which provide interconnected pores facilitating cell attachment and growth. The electrospinning of CP alone, however, is difficult because their backbones are rigid and they exhibit low solubility in solvents. Therefore, they are usually mixed with standard, thermoplastic polymers to achieve materials suitable for electrospinning^8,9^. The coating of independently prepared scaffolds by conducting polymer is another means of preparing conducting 3D structures^10,11^. All of the above-mentioned techniques, however, suffer from an inhomogeneous distribution of conducting components.

Polyaniline-based 3D cryogels are materials swollen with water, which contain a conducting polymer together with a supporting polymer as their constituents^12^. The polymerization takes place in the frozen state and the prefix “cryo” thus refers to the method of preparation; hydrogels are obtained after thawing. Polyaniline is the conducting part of hydrogels, while various water-soluble polymers can be used as the carriers providing the mechanical properties^13^. The utilization of CP-based cryogels is a novel approach affording a solution to the problem of the inhomogeneous distribution of conducting components in the bulk of common conducting hydrogels. It should be noted, however, that research into CP-based 3D materials is in its infancy, and studies dealing with their biological properties are rare despite the fact that cell responses are known to differ on planar and 3D materials.

Polyaniline is generally considered as a polymer with limited biocompatibility^14^. The term biocompatibility refers generally to the ability of a material to coexist with living organisms and tissues without harming them, and its testing can be conducted in a variety of ways. According to the prospective application of the material, defined sets of specific tests are used. As the prepared polyaniline cryogels are considered for applications in bio-sensing and tissue engineering, cytotoxicity, embryotoxicity, stem cell adhesion and growth, and the impact of CP on cardiomyogenesis and neurogenesis have been chosen in order to reveal the basic biocompatibility parameters of this novel and promising material.

## Experimental Sections

The interaction of any material with cells or tissues depends on its surface and bulk properties. The surface energy, pore-size distribution, and elasticity expressed by Young moduli were determined for polyaniline cryogel. In addition, impurities leaching from polyaniline cryogel were characterized by chromatography. These characteristics, together with biological properties, provide a comprehensive view on the applicability of polyaniline cryogel in a variety of applications.

### Preparation of Polyaniline cryogel

Polyaniline was prepared by the oxidation of the respective monomer with ammonium peroxydisulfate^15,16^. Aniline hydrochloride (2.59 g) was dissolved in a 5 wt.% aqueous solution of poly(vinyl alcohol) (PVAL; molecular weight 61,000; Sigma-Aldrich) to 50 mL volume, and ammonium peroxydisulfate (5.71 g) was also dissolved separately in the same solution to the same volume^12^. Both solutions were pre-cooled to 0 °C and mixed, then drawn into polyethylene syringes. The solutions were quickly frozen in a solid carbon-dioxide suspension in ethanol at −78 °C and subsequently placed in a freezer at –24 °C, and aniline was then left to polymerize for 7 days. The concentrations of reactants were 0.2 M aniline hydrochloride, 0.25 M ammonium peroxydisulfate, and 5 wt.% poly(vinyl alcohol)^12^. The originally white ice changed to dark green/black as polyaniline was produced. After thawing, the cryogels were removed^12^ from the syringe and left in water for one week to remove any low-molecular-weight reactants or by-products. The cryogel was composed of ≈2 wt.% polyaniline, 5 wt.% poly(vinyl alcohol), and 93 wt.% water^12^. The cryogel thus has a composite nature where polyaniline affords the conductivity and poly(vinyl alcohol) the mechanical integrity^12^.

### Material Properties

#### Thermal conductivity

The thermal conductivity was measured by TCi Thermal Conductivity Analyzer (C-THERM Technologies, Ltd., Canada) with heat conductivity range 0.01–10 W m^−1^ K^−1^, at 25 °C and instrument regime for porous materials. The cylindrical samples were prepared with diameter 1.5 cm and thickness 0.5 cm.

#### Surface energy

Contact angle measurements and the determination of surface energy were conducted with the aid of the Surface Energy Evaluation System (Advex Instruments, Czech Republic). For polyaniline cryogel, deionized water, ethylene glycol, and diiodomethane (Sigma-Aldrich) were used as test liquids. The droplet volume of the test liquids was set to 2 μL in all experiments, these conducted on a gently dried flat surface of polyaniline cryogel. Ten separate readings were averaged to obtain one representative contact angle. The substrate surface free energy was determined by the “acid–base” method and calculated according to procedure described in work of van Oss^17^.

The acid-base theory enables the determination of the polar and dispersive contributions to the total surface free energy as well as the electron-donor and electron-acceptor components of the polar part of the surface free energy (equation 1).

1 $${\gamma }^{TOT}={\gamma }^{LW}+{\gamma }^{AB},$$

where *γ* ^TOT^ is the total surface energy, the superscript *LW* denotes the total dispersion Lifshitz-van der Walls interaction and *AB* refers to the acid-base interaction. According to Lewis, the acid-base interaction can be determined by equation (2).

2 $${\gamma }^{AB}=2\sqrt{{\gamma }^{+}{\gamma }^{-}},$$

where *γ* ^+^ is the electron-donor and *γ* ^*−*^ is the electron-acceptor component of the acid-base part of the surface energy.

The surface free energy *γ* ^TOT^ can be calculated using Young-Dupré equation (3).

3 $$(1+\,\cos \,{\theta }_{i}){\gamma }_{i}=2(\sqrt{{\gamma }_{i}^{LW}{\gamma }_{j}^{LW}}+\sqrt{{\gamma }_{i}^{+}{\gamma }_{j}^{-}}+\sqrt{{\gamma }_{i}^{-}{\gamma }_{j}^{+}}).$$

Here *j* refers to the studied material, *i* the testing liquid and *Θ* is the measured contact angle.

#### Pore-size distribution

Pore size was estimated by analysis of scanning-electron micrographs of planar sections of freeze-dried polyaniline cryogel by the image analysis. The specific surface of the material was computed based on the assumption that the pores were closed; in reality, pores are partially open, and the real specific surface area is therefore slightly smaller than computed. It should be noted that the pore-size distribution in freeze-dried cryogels may differ somewhat from the pore-size distribution in native hydrogels. For calculation, method of chords recommended in ASTM E112-13 standard was employed. The system of random lines was drawn to planar section of image from which the lengths of individual chords were measured by automatic image analysis. From the mean chord length the mean pore size was subsequently estimated. For pore size estimation 10 images with magnification of 1000x were used and about 500 of chords were measured on each image.

The porosity was computed as 1−*V*_d_/*V*_w_, where *V*_d_ is the volume of the polymer estimated from the mass density of the polymer and the mass of the dry sample, *V*_w_ is the total volume of the sample computed from the geometric parameters of the cylindrical sample and independently of the mass of the wet sample (difference was less than 5%).

#### Young modulus

Young modulus was determined on a Shimadzu Autograph AG-X tensile tester. The analyses were performed on four cylindrical samples with a length 10 mm and a diameter 9.2 mm. Each sample was inserted between two horizontal plates and deformed with a rate of 1 mm min^−1^. Measurement time was of 3 min and within this short period of time the humidity of the sample was considered constant. The Young modulus was determined as a slope of the liner part of the stress-strain curve. The experiments were performed in compression.

### Impurity profile

Concentrations of residual impurities were determined using a modular HPLC system consisting of a Waters 600E pump, a VD 040 vacuum degasser (Watrex, Czech Republic), and a UV200 ultraviolet detector (Watrex, Czech Republic). A reversed-phase C18 column X-select (300 mm × 7.8 mm; Waters) was employed. The analysis was performed in isocratic mode with an acetonitrile/acetate pH 4 buffer at a ratio of 60/40 (v/v) as the mobile phase. A flow rate of 0.8 mL min^−1^ and 20 μL injection volume were employed. Analytes were monitored at 235 nm by the UV detector. Data acquisition and analysis were performed using a Clarity Chromatography Station. For HPLC analysis, polyaniline samples were extracted in accordance with ISO 10993-12 in the ratio of 0.1 g of polyaniline cryogel per 1 mL of ultrapure water.

### Biocompatibility

The study was approved by Committee on Animal Research and Ethics Faculty of Medicine Masaryk University.

#### Used cell lines

Primary mouse embryonic fibroblasts (MEF) were used to determine the cytotoxicity of individual extracts. They were isolated from 13.5 days post coitum (dpc) mouse embryo, mouse strain C57BL/6. Mice were obtained from the Laboratory Animal Breeding and Experimental Facility of the Faculty of Medicine, Masaryk University, Brno, Czech Republic and kept under controlled conditions; standardized diet pellets and UV light-treated tap water were available *ad libitum*. Experiments were performed in the accordance with national and international guidelines on laboratory animal care and with the approval of the Institute Ethical Committee. Embryos were washed in Phosphate Buffered Saline (PBS) and decapitated, and the inner organs removed. For the separation of fibroblasts, the embryos were trypsinized and mechanically disrupted by pipeting. A single-cell suspension was seeded onto a tissue culture dish in complete high glucose Dulbecco’s modified Eagle’s medium (DMEM) supplemented with 100 U mL^−1^ of penicillin, 0.1 mg mL^−1^ of streptomycin, 15% Foteal Bovine Serum (FBS) (all from Invitrogen-Gibco) and 0.05 mM 2-mercaptoethanol (Sigma-Aldrich). The first seeded MEFs were designated as passage 0. In our experiments, only MEFs up to passage 3 were employed.

The *embryonic stem cell ES R1* line^18^ was propagated in an undifferentiated state by culturing on gelatinized tissue culture dishes in complete DMEM media. The gelatinization was performed using 0.1 wt% porcine gelatin in water. Dulbecco’s Modified Eagle’s Medium containing 15% fetal calf serum, 100 U mL^−1^ penicillin, 0.1 mg mL^−1^ streptomycin, 100 mM non-essential amino acids (all from Gibco-Invitrogen; USA), 0.05 mM 2-mercaptoethanol (Sigma Aldrich) and 1 000 U mL^−1^ of leukemia inhibitory factor (Chemicon; USA) was used for the cultivation.

Preparation of ESc R1 line-derived cardiomyocytes from the HG8 clone were described previously^19,20^. Purified cardiomyocytes were seeded onto reference tissue culture dishes and onto all tested polyaniline surfaces. DMEM:F12 (1:1) supplemented with 5% fetal calf serum, antibiotics as above, and insulin, transferrin, and selenium supplements (ITS; all from Gibco-Invitrogen) was used as the growth medium.

*Neural progenitors* were isolated from ganglionic eminences of embryonic brain (13.5 dpc)^19,21^ and expanding in neurobasal media DMEM:F12 with B27 and N2 supplements, 100 U mL^−1^ penicillin, 0.1 mg mL^−1^ streptomycin (Gibco-Invitrogen; USA), 5 ng mL^−1^ of FGF-2 and 20 ng mL^−1^ of EGF (PeproTech; USA). Only cells up to passage 3 were used.

#### Cytotoxicity and embryotoxicity

The cytotoxicity testing of extracts of polyaniline cryogel was performed according to ISO 10 993-5. Samples were extracted according to ISO 10993-12 in the ratio of 0.2 g mL^−1^ of relevant cultivation medium. Extraction was performed in chemically inert closed containers using aseptic techniques at 37 ± 1 °C under stirring for 24 ± 1 h. The parent extracts (100%) were then diluted in a culture medium to obtain a series of dilutions with concentrations of 50, 25, 10, 5 and 1%. All extracts were used within 24 h. Cells were pre-cultivated for 24 h and the culture medium was subsequently replaced with polyaniline extracts. As a reference giving 100% cell proliferation, cells cultivated in the pure medium were used. To assess cytotoxic effects, the MTT assay (Invitrogen Corporation, USA) was performed after one-day cell cultivation at 37 ± 0.1 °C. The absorbance was measured at 570 nm by an Infinite M200 PRO (Tecan, Switzerland). All tests were performed in quadruplicates. Dixon’s Q test was used to remove outlying values. The morphology of the cells was observed using an inverted Olympus phase contrast microscope (Olympus IX81, Japan).

Polyaniline cryogel embryotoxicity was analysed as the likelihood of the formation of beating foci within spontaneous differentiating ES R1 cells carrying Nkx2.5-GFP reporter construct (NKX2-5-Emerald GFP BAC reporter), which mediated cardiomyocyte specific expression of green fluorescent protein (GFP) was used^22^. An 5-day-old EBs^20,23^ derived from ES R1 cells clone NK4 were seeded onto polyaniline cryogel or tissue culture plastics coated with gelatin. After 13 days (day 18 of overall differentiation), the appearance of both GFP positive cells and beating foci was checked.

#### Cytocompatibility

*Adhesion and growth of stem cells*: ES R1 cells were seeded onto tissue culture plastics (reference) or onto polyaniline cryogel at a density of 4 × 10^4^ cells per cm^2^. They were uploaded by calcein AM (10 μM; Invitrogene) or after ES R1 cell growth for two days, then fixed by 2% formaldehyde and visualized through nuclei staining by 4′,6-diamidine-2′-phenylindole dihydrochloride (DAPI; 10 ng mL^−1^, Sigma). The number of ESc was quantified by counting of viable cells (counterstained by calcein) visible on photomicrographs.

*Adhesion and expansion of embryoid bodies* (*EBs*): 5-day-old EBs were seeded onto tissue culture plastic (reference) or onto polyaniline cryogel. On day 20 of differentiation overall, expanding EBs were counterstained by calcein AM (10 μM, Invitrogene).

*Adhesion and growth of cardiomyocytes*: Purified ES-derived cardiomyocytes were seeded onto tissue culture plastics (reference) or onto polyaniline cryogel. After two days, cells were fixed by 2% formaldehyde and counterstained by antibody against cardiomyocite specific myosine heavy chain (MF20 antibody, developed by Donald and Fischman, was obtained from the Developmental Studies Hybridoma Bank developed under the auspices of the National Institute of Child Health and Human Development and maintained by the University of Iowa, Department of Biological Sciences). Anti-mouse IgG antibody conjugated to Alexa 568 fluorochrome (Invitrogen) was used as the secondary antibody. Cell nuclei were counterstained by DAPI (10 ng mL^−1^, Sigma)^20^. When the visualization of viable cardiomyocytes was required, the ES R1 cell clone NK4 carrying Nkx2.5promotor-GFP reporter (RP11-88L12 NKX2-5-Emerald GFP BAC Reporter, from BACPAC Resources Children’s Hospital and Research Center at Oakland), which is specifically expressed only in cardiomyocytes^24^, was used^20^. The number of cardiomyocytes was quantified by counting of viable cells (counterstained by calcein) visible on micrographs.

*Neural progenitors*: Neural stem/progenitors cells (NSCs) were isolated from the embryonic ganglionic eminence (GE) of the forebrain of C57/BL6 mice at 13.5 dpc. C57BL/6 mice were obtained from the Laboratory Animal Breeding and Experimental Facility of the Faculty of Medicine, Masaryk University, Brno, Czech Republic. The mice were kept under controlled conditions; a standardized pelleted diet and HCl or UV light-treated tap water were available *ad libitum*. Experiments were performed in accordance with national and international guidelines on laboratory animal care and with the approval of the Institute’s Ethical Committee conforming to the guidelines from Directive 2010/63/EU of the European Parliament on the protection of animals used for scientific purposes^21^.

Three-day-old neurospheres were seeded onto tissue culture plastic with gelatin coating or onto polyaniline cryogel. Neurosphere differentiation was mediated by the withdrawal of neural supplements (B27, N2) and growth factors (EGF, FGF-2), and by supplementation with 2% bovine serum. Differentiating cells were stained by calcein AM (10 μM) or fixed by 2% formaldehyde, and F-actin was stained by phaloidin-FITC (Sigma-Aldrich). Nuclei were counterstained by DAPI (Sigma-Aldrich, 10 ng mL^−1^). Cell pictures were taken using an Olympus digital camera (E-450) mounted onto an Olympus inverted microscope (IX51). The number of neural progenitors was quantified by counting of viable cells (counterstained by calcein) present on micrographs.

A fluorescent pictures or videos of cells were taken using an Olympus E-450 digital camera (photos) or INFINITYLite camera (videos) mounted onto an Olympus inverted epifluorescent microscope IX51.

## Results and Discussion

### Material Properties

Materials properties of cryogel can be classified into surface and bulk properties. While surface properties are crucial with respect to the first contact of a material with biological fluids and cells, bulk properties, such as porosity and elasticity, play a crucial role in its long-term interaction with cells and tissues. The coefficient of thermal conductivity for swollen cryogel sample was (1.3 ± 0.1) W m^−1^ K^−1^.

Surface energy, as a basic surface characteristic of polyaniline cryogel is shown Table 1. The obtained values indicate that the surface of the cryogel is extremely hydrophilic as the only value of contact angle, which can be taken into consideration refers to the methylene iodide. The other liquids created sessile drops below the adequate level. This result is in agreement with expected data for PVAL systems. Cell adhesion is a process that is affected by the properties of the surfaces to which the cells adhere. These include a broad range of characteristics such as topography, porosity wettability, softness, roughness microstructure as well as presence of characteristic functional groups on the material surface^25^. Key role in the cell response plays protein adsorption, which exhibits the first step preceding attachment of cells. Thanks to their amphiphilic character, proteins can absorb on both hydrophilic and hydrophobic surfaces. On the hydrophobic surfaces they adsorb through hydrophobic patches present on their surface. In the case the surfaces are hydrophilic, they interact with the polar and charged functional groups^26^. Hence the protein composition and conformation are critical characteristics with respect to subsequent cell adhesion and influence also nature of cells to be adsorbed on the surfaces. Detailed description of mechanism of adsorption of proteins on polymers surface is beyond the scope of the current manuscript. However taking into account the published studies the hydrophilicity of the polymer is considered preferable for adsorption of the cells^27^.

**Table 1:** The surface energy of polyaniline cryogel.

*γ* ^tot^ (mN m^−1^)	*γ* ^LW^ (mN m^−1^)	*γ* ^AB^ (mN m^−1^)	*θ* ^W^ (deg)	*θ* ^E^ (deg)	*θ* ^M^ (deg)
47.6	20.5	27.1	1.66 (<15)	1.99 (<15)	74.2

Total surface energy, *γ* ^*tot*^, dispersive component of surface energy, *γ* ^*LW*^, acid–base component of surface energy (polar), *γ* ^*AB*^, and contact angles, θ, determined using deionized water (W), ethylene glycol (E), and methylene iodide (M) as wetting agents.

**Figure 1:**
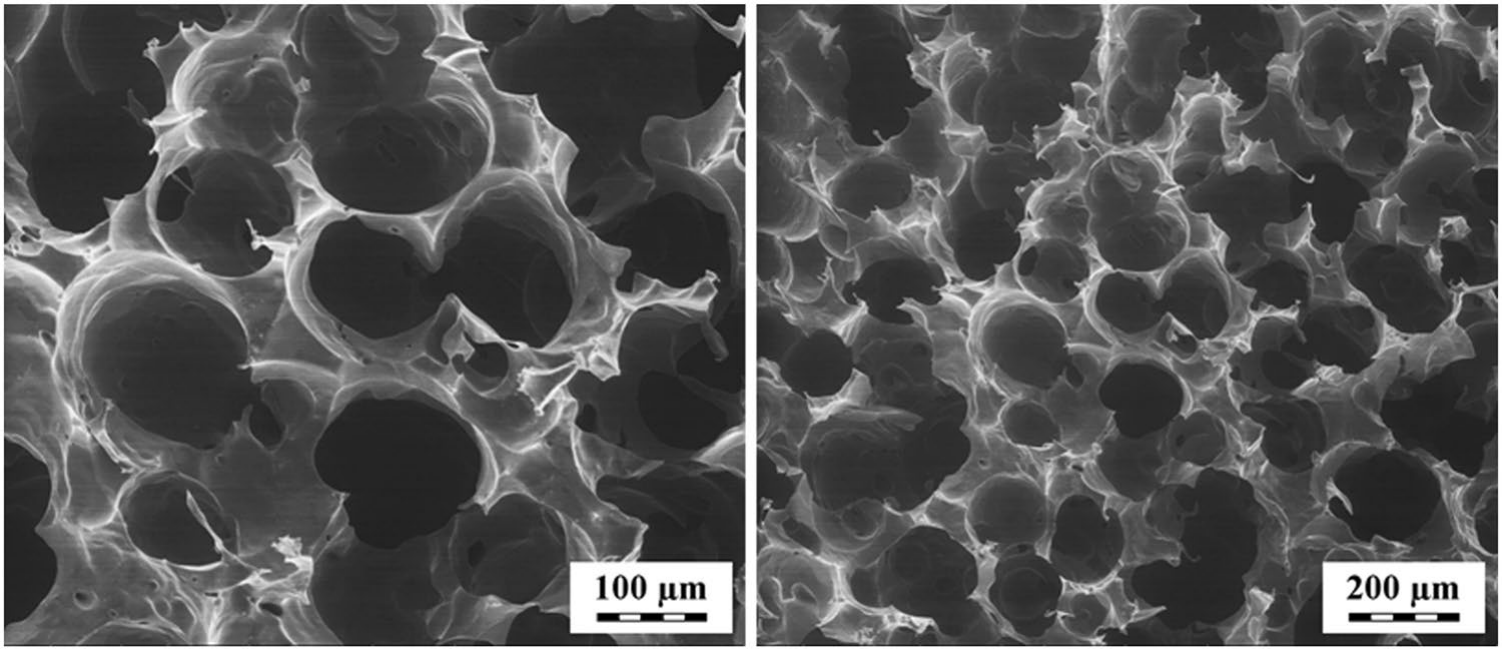
Scanning electron micrographs of cryogel (2 magnifications).

Porosity is a crucial factor not only influencing the ability of cells to migrate and grow within the structure but also providing biomechanical stimuli and influencing the microenvironment (e.g., with respect to the release of biofactors or efficient nutrient exchange). Moreover, porosity affects vascularization and facilitates mechanical interlocking between scaffolds and surrounding tissue^28^. As can be seen from the scanning-electron micrographs (Fig. 1), polyaniline cryogel has a highly macroporous structure. The mean pore size obtained from the image analysis of planar sections of cryogel was estimated to 159 μm and corresponding specific surface area to 0.020 m^2^ cm^−3^. Pore size distribution was not computed; however, from images it can be concluded that it is quite narrow and covers sizes from tens to hundreds of microns.

The porosity of sample estimated from comparison of swollen and dry mass of cryogels reveals value of 95.5 vol%. Therefore, polyaniline cryogel meets the criteria for scaffolds.

**Figure 2:**
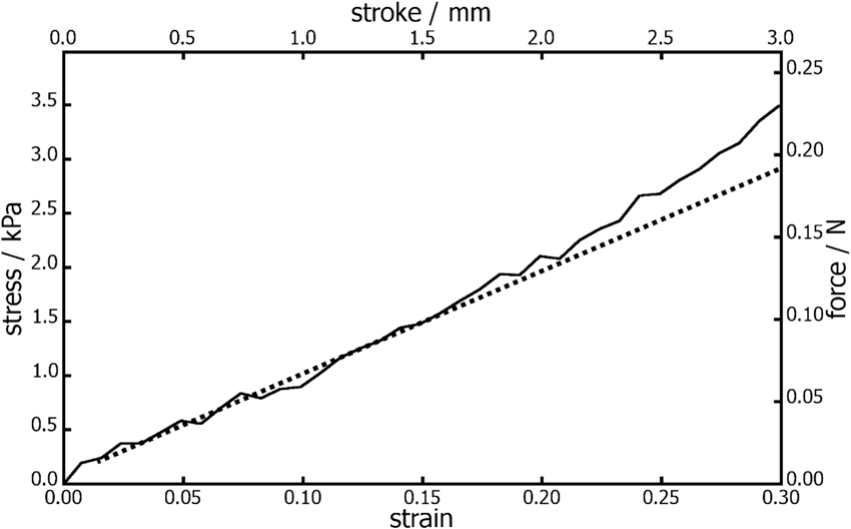
Stress-strain curve used for calculation of Young modulus of PVA-PANI cryogel measured under confined conditions. The experiments were performed in compression.

**Figure 3:**
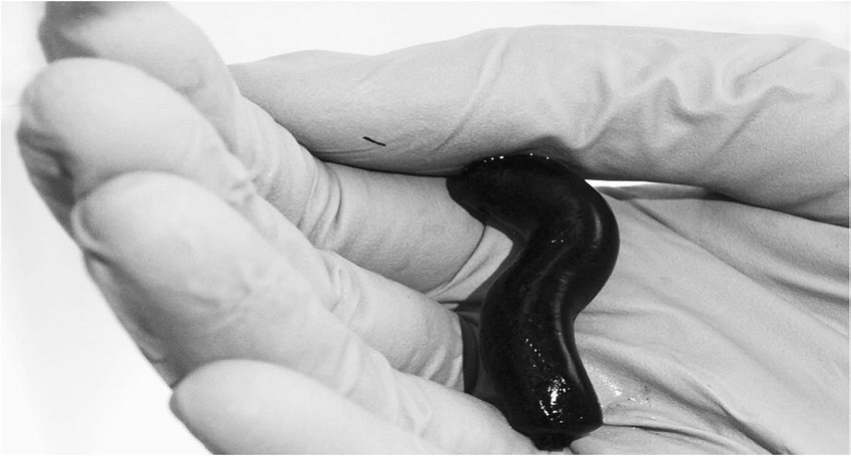
Polyaniline cryogel mimicking the properties of native tissue. Polyaniline is green in transparent thin films but appears black in thick layers or in powder form^16^.

Elasticity is the second important bulk property influencing tissue reactions to the scaffold. Metallic neural interfaces can serve as a good example as their mechanical and structural properties are highly dissimilar to those of neuronal tissue, a situation which can lead to irritation and adverse immune responses^1^. Polyaniline cryogel has properties much closer to those of native soft tissues than for example mentioned metallic devices. The mean value of Young modulus of polyaniline cryogel was determined to (9.7 ± 0.5) kPa; the example of stress-strain curve recorded on the cryogel is given in Fig. 2. With respect to obtained value of Young modulus and the fact that typical soft tissues have a Young modulus ≈1 MPa^29^ or even lower, it can be concluded that the cryogel exhibits properties of elastic material. Moreover, the elasticity of swollen polyaniline cryogel is demonstrated in Fig. 3.

### Impurity Profile

With respect to the preparation procedure, residual aniline hydrochloride and ammonium peroxydisulfate are the main expected impurities in polyaniline cryogel. Both of these substances are considered as potentially harmful^30,31^. Chromatographic analyses revealed that the concentrations of residual aniline hydrochloride and ammonium peroxydisulfate in native polyaniline cryogel were 12.8 ± 0.5 μg g^−1^ and 2.3 ± 0.3 mg g^−1^ of the cryogel, respectively. In addition to these two impurities, two other unknown peaks were observed on chromatogram. With the aid of appropriate standards and their retention times, these two peaks were identified to be oxidation products of aniline, namely hydroquinone and *p*-benzoquinone^32^. Their concentrations were of 8.4 ± 0.3 μg g^−1^ and 36 ± 3 mg g^−1^ of the cryogel, respectively.

**Figure 4:**
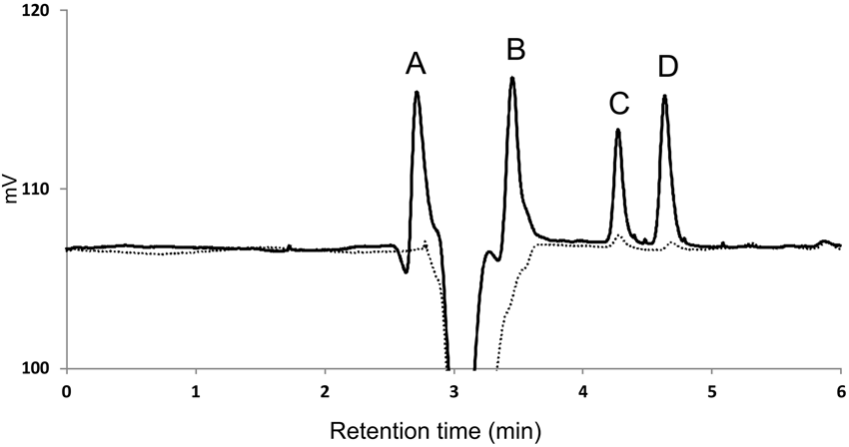
HPLC chromatogram of impurities detected in extracts of native (solid line) and purified (dotted line) polyaniline cryogels. Residuals of A – ammonium peroxydisulfate, B –hydroquinone, C – *p*-benzoquinone, D – aniline hydrochloride.

Chromatogram of impurities detected in cryogel extract is presented in Fig. 4. It can be assumed that the cryogels also contain residual ammonium sulfate originating from polymer synthesis^11,15^. However, this impurity can’t be detected by UV detector and, moreover it falls to a group of substances generally recognized as safe (GRAS) by U.S. Food and Drug Administration^33^ and hence it is not expected to have any significant harmful effect to the cells. To reveal if the additional purification can lead to elimination of low molecular impurities the polyaniline cryogel was further purified by the repeated extraction of 5 g of cryogel with 50 mL ultrapure water for 24 h until a pH 7 of the extract was achieved. Additional purification of the sample through its extraction with ultrapure water further reduced the content of impurities leached from it. The concentrations of aniline hydrochloride and *p*-benzoquinone were lower than 1 μg g^−1^ of cryogel and ammonium peroxydisulfate and hydroquinone were not detected. Additionally purified cryogel was used only for determination of impurities and cytotoxicity. All the other tests, however, were performed on native cryogel without additional purification not to bias the biocompatibility of native polyaniline cryogel.

### Biocompatibility

#### Cytotoxicity and embryotoxicity

Cytotoxicity is the basic parameter of biocompatibility, which demonstrates the ability of cells to survive in the presence of foreign materials – in present case, the extracts of the tested polyaniline cryogel. It is known that polyaniline powder displays significant toxicity^14,19^. Cytotoxicity is mainly connected with the presence of low-molecular-weight impurities^34^. Cryogel contains of 2 wt.% of polyaniline in the matrix; thus its toxicity should be lower than in the case of polyaniline powders, which was confirmed in the current work.

**Figure 5:**
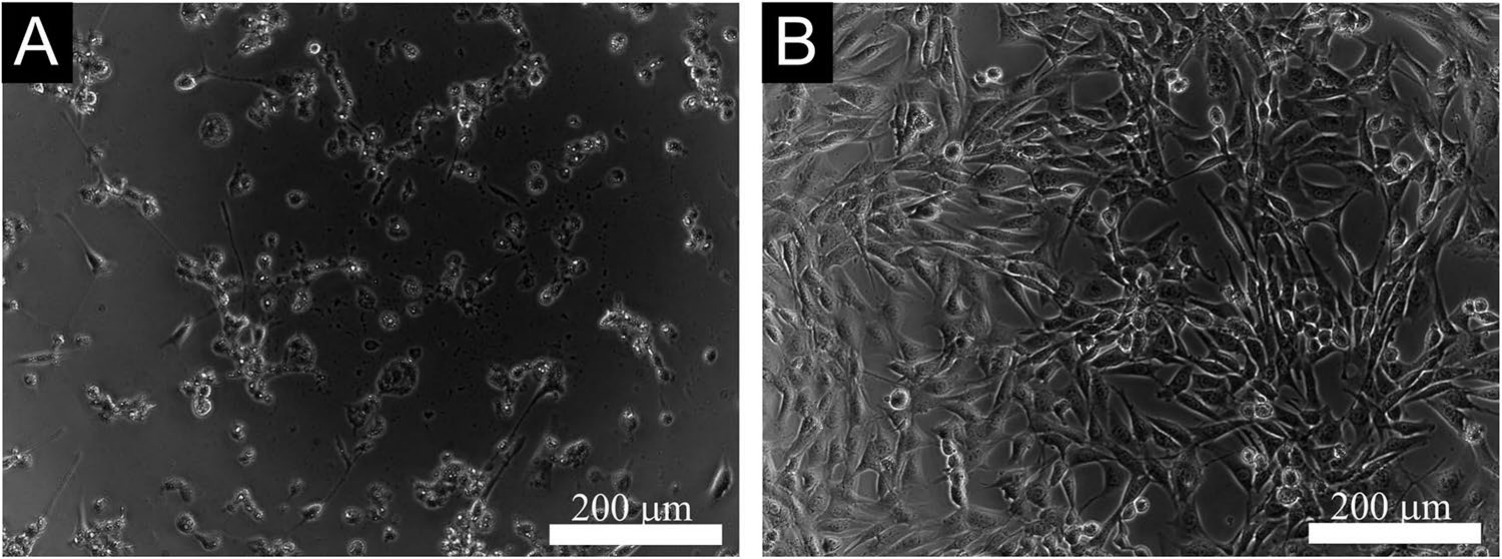
Cytotoxicity of extracts of native (**A**) or purified (**B**) polyaniline cryogel determined as relative number of viable MEF cells cultivated in the presence of extracts for 24 h. The dashed lines highlight the limits of viability according to EN ISO 10993-5: viability >0.8 corresponds to no cytotoxicity, >0.6–0.8 mild cytotoxicity, >0.4–0.6 moderate toxicity and <0.4 severe cytotoxicity.

As described above, the cytotoxicity tests on MEF were performed not only on native but also on additionally purified polyaniline cryogels. Cell viability was similar for native and purified samples, and 5, 10, 25, 50 and 75% extracts caused no cytotoxicity (cell survival was higher than 80%, compared to the reference). Only the parent 100% extracts showed mild cytotoxicity (cell survival 60 to 80%). The cytotoxicity of extracts is clearly illustrated in Fig. 5. It can be concluded that although purification leads to a decrease in the amount of low-molecular-weight impurities present in polyaniline cryogel, the impact of washing on cytotoxicity is negligible. In all the other tests, the native non-purified polyaniline cryogel was used to demonstrate the properties of native polyaniline cryogel as a new material.

**Figure 6:**
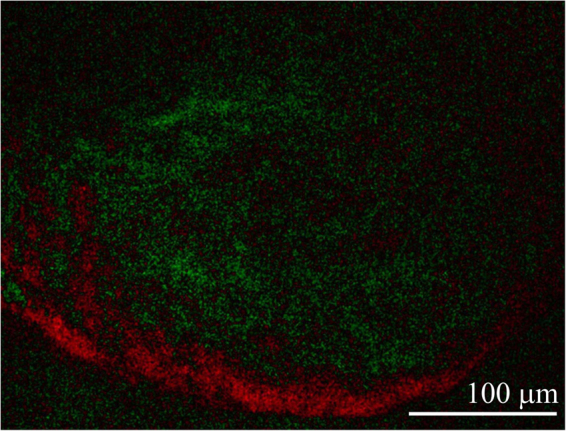
Beating foci within spontaneous differentiating ESc on polyaniline cryogel. The difference between microscopy images taken with a 0.3 s time shift. Red spots correspond to the movement of cells to the right. Green spots indicate no difference between the images (see the supplement for video of the beating foci). The video was taken on day 18 of differentiation overall.

The appearance of beating cardiomyocytes in EB outgrowths is used as one of toxicological end-points to assess the embryotoxic potential of tested samples. In this experiment, beating foci within spontaneous differentiating ES R1 cells through formation of EBs were observed both on tissue culture plastics and on polyaniline cryogel, the foci beating in the same manner and frequency. The beating is presented in micrographs of beating foci on polyaniline cryogel (Fig. 6) and in a supplementary video. Combining the results of cytotoxicity testing on differentiated fibroblasts with visual assessment of the morphology of beating cardiomyocytes in differentiating embryonic stem cells is considered as a standard procedure for the evaluation of embryotoxicity^35^. It can therefore be concluded that polyaniline cryogels do not exhibit embryotoxicity.

#### Cyto-compatibility

The application of any material in regenerative medicine, tissue engineering, or biomedicine presumes that its surface will be in contact with cells. Although the adhesion of cells on surfaces *in vivo* depends on the adhesion of proteins, which can significantly alter the surface properties of the material, the adhesion of cells under *in-vitro* conditions is the technique generally accepted for evaluating the interaction between surfaces and cells. It is well known that cell types require different surface properties for their growth. Therefore, the responses of cells related to electro-sensitive tissues were tested on polyaniline cryogel. Namely, stem cells, embryoid bodies, cardiomyocytes, and neural progenitors were used.

**Figure 7:**
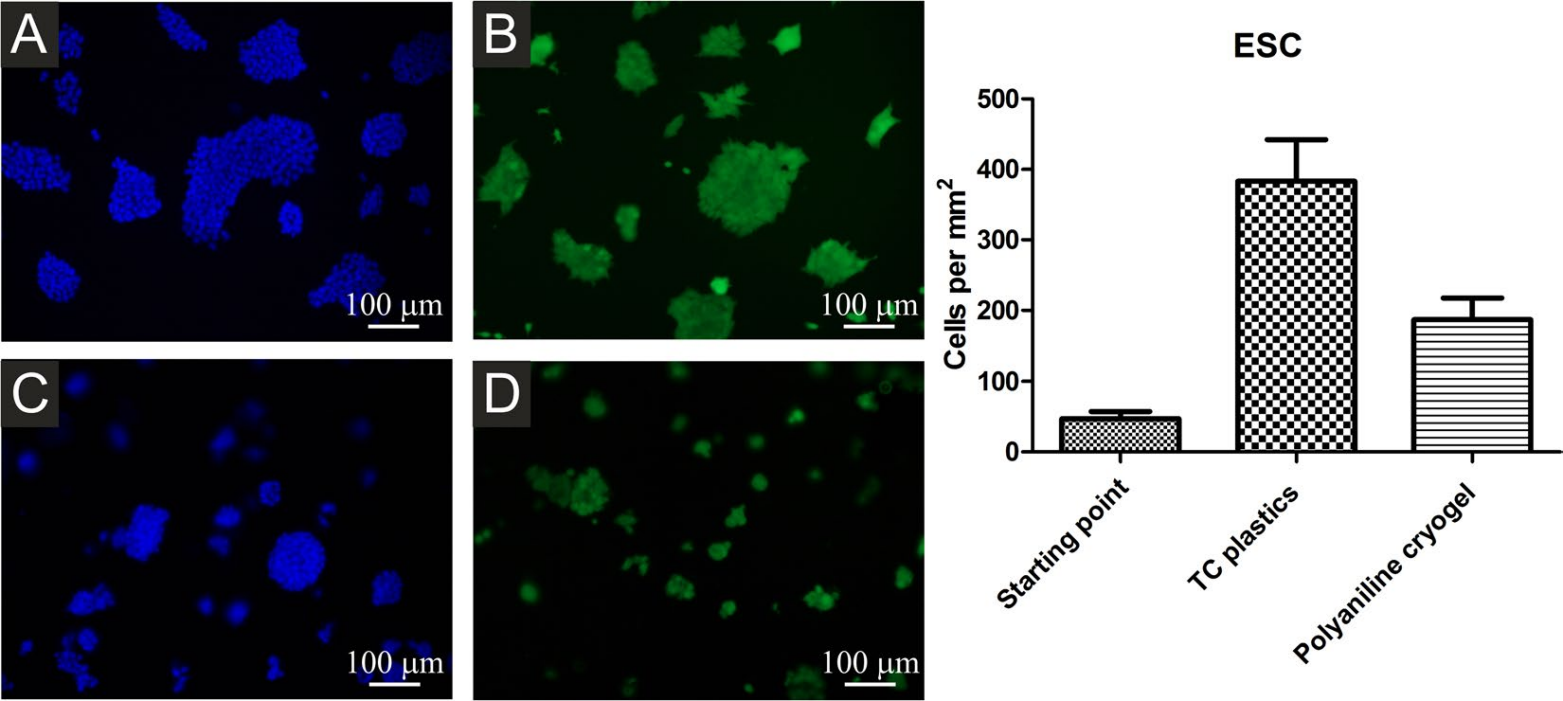
ESc growing on TC plastics (**A**,**B**) or polyaniline cryogel (**C**,**D**). Viable cells are visualized by calcein AM (**B**,**D**), all cells through nuclei counterstain by DAPI (**A**,**C**). Starting point is defined as a number of adherent cells 4 h hours after seeding. The ESc adhered and proliferated on the cryogel to a lesser extent than on the TC plastic. The micrographs were taken on day 2 after seeding.

Stem cells are generally considered for application in regenerative medicine and tissue engineering. In general, ES R1 cells were able to adhere, grow and form compact colonies on tissue culture plastics (Fig. 7A,B). A slightly different situation arose on the surface of polyaniline cryogel. Thanks to DAPI counterstaining, which visualizes the nuclei of cells (Fig. 7C,D), it can be seen that the number of stem cells on the cryogel surface was lower in comparison with the reference. This was confirmed by counterstaining with calcein which determined only viable cells (Fig. 7). On the cryogel, ES R1 cells formed more compact colonies, which was probably the result of their lower capacity to adhere to its surface. This was also demonstrated by the presence of more compact colonies and also by the smaller number of viable cells.

**Figure 8:**
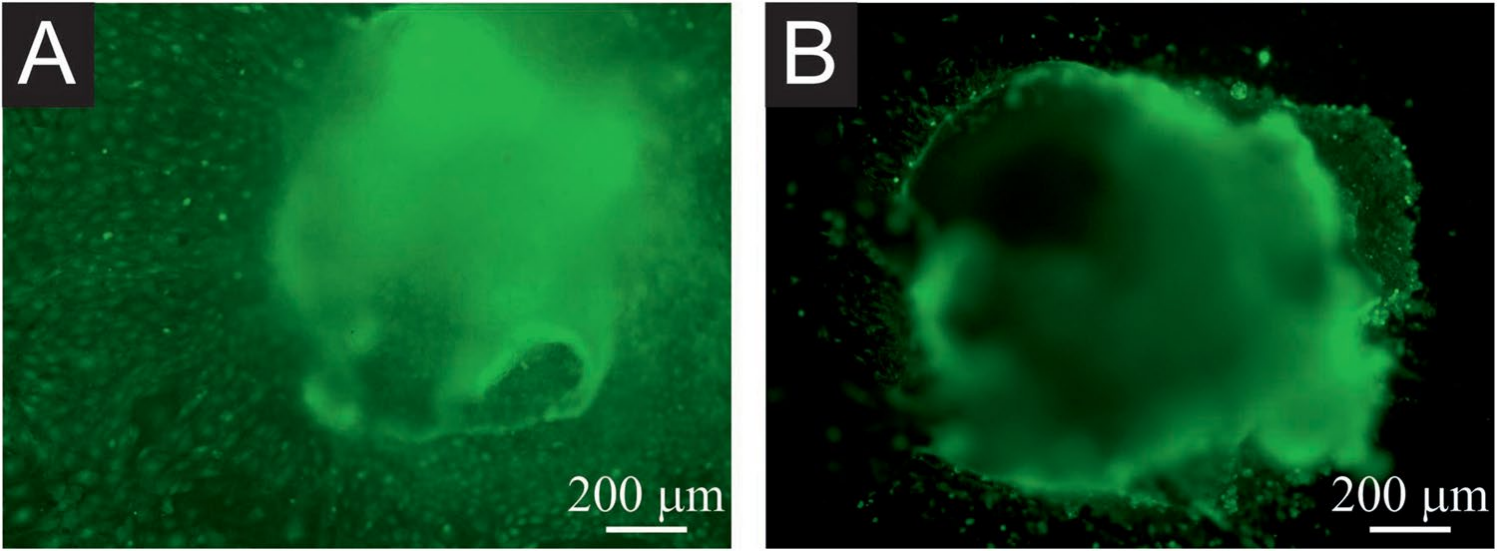
EB growth on TC plastics (**A**) and polyaniline cryogel (**B**). Only viable cells are visualized by calcein. The migration of cells (represented by individual spots) is weaker on polyaniline cryogel (**B**) than on TC plastic. The micrographs were taken on day 20 of differentiation overall.

A similar situation was encountered after previously formed EBs were seeded on the polyaniline cryogel (Fig. 8). After the seeding the EBs either attached on the surface or stayed floating. The number of attached and floating EBs were similar in case of TC plastic or polyaniline cryogel. The floating EBs were removed when cultivation medium was changed. From the EBs which attached to the surface, the cells can further migrate and proliferate. As can be seen from Fig. 8A,B, where only viable cells are visualised, the migration and proliferation of cells from EBs is more intense in case the EBs are attached to TC plastic than to polyaniline cryogel. This is depicted by the green spots visible outside the EBs which are present in higher quantity on TC plastic than on cryogel. EBs seeded onto polyaniline cryogel maintained their compact morphology better, and cell growth outside EB boundaries was relatively rare (Fig. 8B). Comparison of cell behaviour visualized in Fig. 8A and B indicates that polyaniline cryogel allows for the adhesion of EBs, but the migration is less intensive than on reference TC plastics.

**Figure 9:**
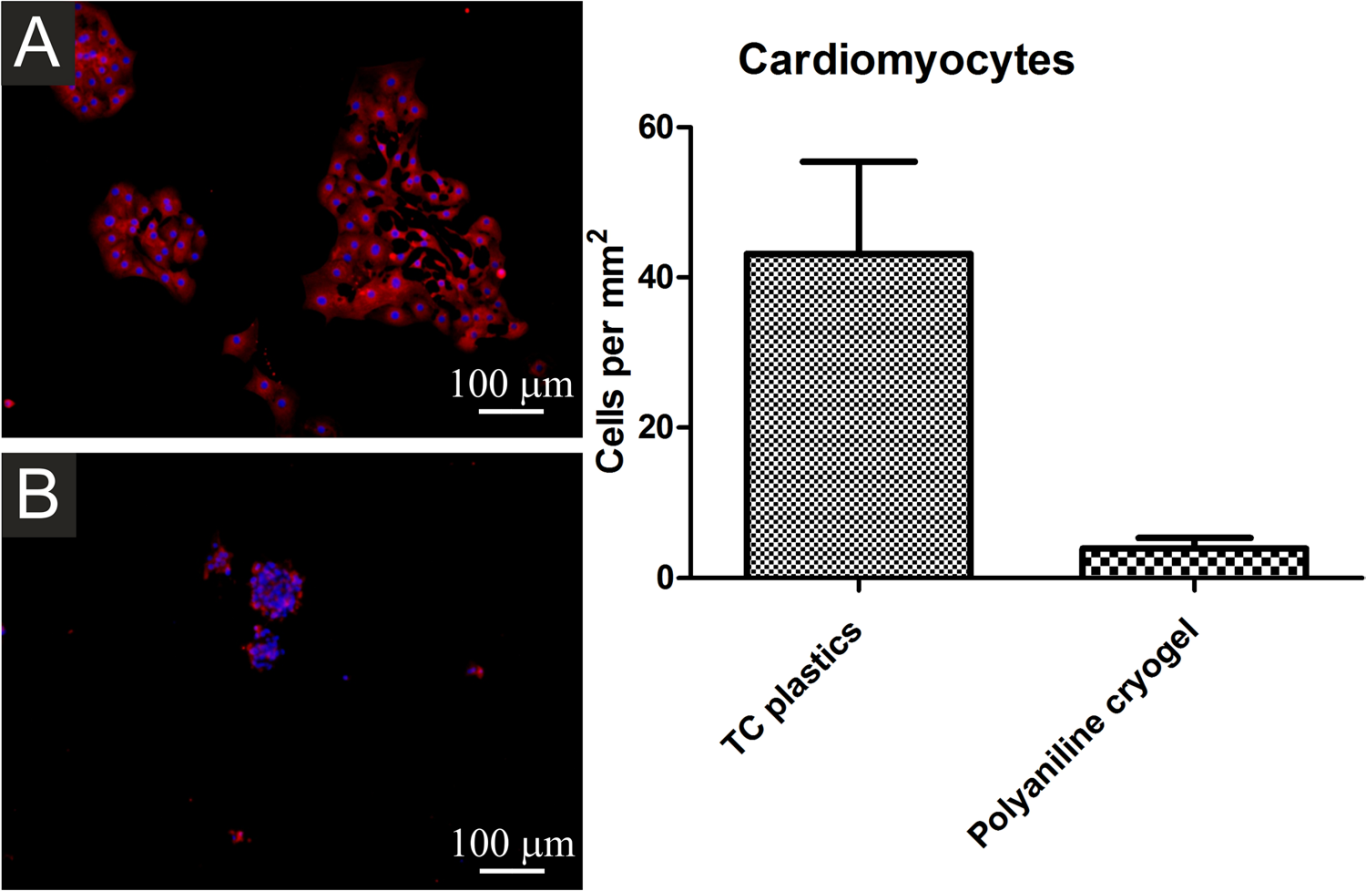
Isolated cardiomyocytes seeded onto TC plastics (**A**) and polyaniline cryogel (**B**). Cardiomyocytes were visualised using antibody against cardiomyocyte-specific myosine heavy chain (red). Individual cells were visualised through nuclei counterstaining by DAPI (blue). Number of cells at the starting point is not presented as the cardiomyocytes did not proliferate after the seeding. The number of cardiomyocytes adhered on the cryogel was significantly smaller than on TC plastic. The micrographs were taken on day 2 after seeding.

The weakest interaction between the cryogel surface and cells was observed in the case of cardiomyocytes and neural progenitors. Cardiomyocytes seeded onto TC plastics adhered well to this surface. In contrast, they were not able to adhere to polyaniline cryogel (Fig. 9). The differences in the responses of ES R1 cells, EBs, and cardiomyocytes can be explained by the specific properties of cardiomyocytes. In comparison with the other types of cells used, cardiomyocytes were already beating when they came into the contact with the surface of the cryogel. In general, such beating can have an impact on their ability to adhere to surfaces. The behavior of EBs, however, shows that cardiomyocytes can spontaneously differentiate within the EB on the cryogel. It can therefore be expected that spontaneously differentiating cardiomyocytes will probably be able to grow on the cryogel surface.

**Figure 10:**
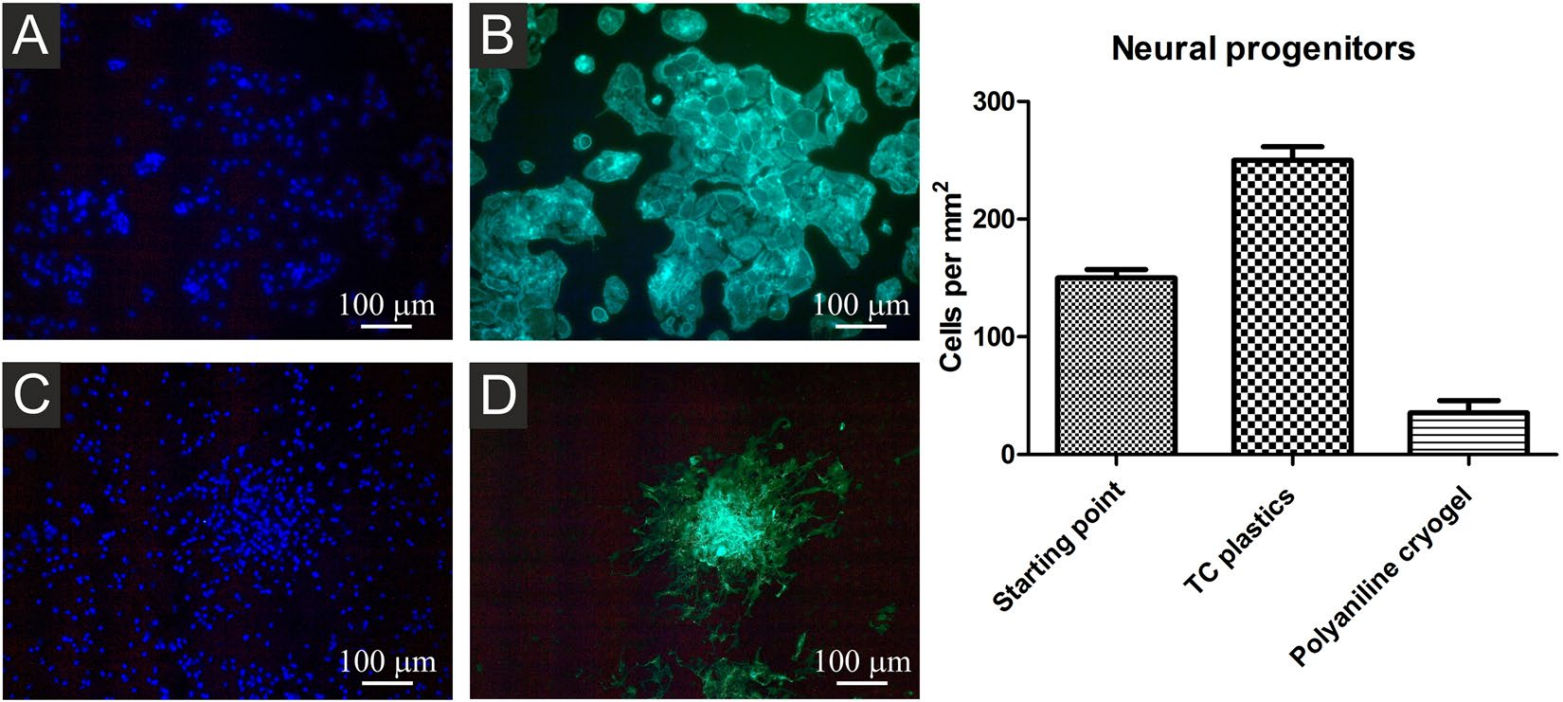
Neural progenitors differentiating on TC plastics (**A**,**B**) and on polyaniline cryogel (**C**,**D**). Cells were visualized by F-actin detection using DAPI  (**A**,**C**). Cell nuclei were counterstained by phalloidin FITC (**B**,**D**). Starting point is defined as number of adherent cells 12 h hours after seeding. The number of adhered neural progenitors on the cryogel was significantly lower than on TC plastic, thus also the proliferation of neural progenitors on the polyaniline cryogel is low. The micrographs were taken on day 4 of differentiation overall.

Differentiating neural progenitors were seeded onto TC plastics or polyaniline cryogel and visualized using phalloidin-FITC (binding to F-actin), or by calcein AM (determined viable cells). The micrographs show that neural progenitors formed compact colonies of well-spread cells on tissue plastic both with and without gelatin coating (Fig. 10A,B). When seeded onto polyaniline cryogel, rare colonies of poorly-spread cells were observed (Fig. 10C,D). Corresponding results were also observed for cells stained with calcein AM, which visualizes only viable cells (not shown).

Any polymer intended for application in regenerative medicine or biosensors must fulfil a number of criteria including good biocompatibility, the presence of appropriate bulk and surface properties and, in particular cases, also the presence of more specific properties, such as conductivity. Some of these properties can be achieved through the use of conducting polymers; however, such polymers do not possess appropriate bulk properties as they can only be prepared in the form of thin films (in case of polyaniline 40–400 nm thick)^36^, powders, and colloidal dispersions, which all have only limited application potential. Due to these shortcomings, it is advantageous to combine conducting polymers with other polymer biomaterials. The efficacy of this strategy has recently been confirmed by a number of research studies^9,37–40^. Here, the bulk properties of the studied polyaniline cryogel correspond to those of hydrogels and are ideal for contact with soft tissues. One of the advantages of using polyaniline is the ability to modify its surface properties by a variety of simple methods including reprotonation with various acids, the grafting of functional groups, and copolymerization with various co-monomers, etc. It is also well known that any type of cell requires specific surface properties, as was unambiguously confirmed by the different behaviours of PC12 cells on polyaniline-grafted with bioactive peptides^41^. The limited ability of ESc, EBs, cardiomyocytes, and neural progenitors to adhere, grow and proliferate on polyaniline cryogel corresponds to previously published findings^41^ and confirms that polyaniline-based materials require post-preparation surface modification, which is tailored to the specific cells used in order to improve such materials’ cyto-compatibility.

Polyaniline cryogel is a new form of biomaterial. The purpose of present study is therefore to describe basic biological properties of its native form. In context of future studies, the cytotoxicity and embryotoxicity of native polyaniline cryogel is more important than surface properties influencing the cell adhesion, proliferation and migration, as surface properties can be easily modified by various techniques to achieve the desired interaction with concrete cell lines. Polyaniline cryogels combine poly(vinyl alcohol) and conducting polyaniline. To determine the cytotoxicity of polyaniline, it is best to study polyaniline powder, as it has the highest content of potentially hazardous components compared to thin films or colloidal dispersions. A previously published study dealing with the biocompatibility of standard powder polyaniline hydrochloride prepared by the IUPAC-approved procedure^15,16^ through the oxidative polymerization of aniline hydrochloride with ammonium persulfate indicated that cytotoxicity can be related to low-molecular-weight compounds accompanying the polymer^14^. A reduction in cytotoxicity was observed after purification procedures aimed at the removal of impurities found in pristine powder polymer. Whether such procedures involved reprotonation/deprotonation^14^, reprecipitation^42^ or Soxhlet extraction^34^, all such purification methods pointed to residual monomers or low-molecular-weight by-products as being responsible for cytotoxicity. In standard polyaniline powder, impurities related to residual precursors used for polymerization, i.e. aniline hydrochloride and ammonium peroxydisulfate, were determined in the respective extracts. HPLC analyses showed that polyaniline hydrochloride polymer leached out residual aniline hydrochloride and ammonium peroxydisulfate in concentrations of 0.95 ± 0.03 mg g^−1^ and 96.1 ± 1.9 mg g^−1^ of polymer powder, respectively. The sample exhibited cytotoxicity against two different cell lines, human immortalized non-tumorogenic keratinocyte cell line (HaCaT) and human hepatocellular carcinoma cell line (HepG2). In both cases, the cytotoxicity was dependent on the concentration of impurities in the extract and the type of cells to which the extract was applied. When graded according to the requirements of EN ISO 10993-5, the cytotoxicity of parent 100% extract of polymer was assigned as severe for HaCaT cells (a survival rate lower than or equal to 40%) and moderate for HepG2 (a survival rate of 40–60%). After the parent extract was diluted, the first entirely non-cytotoxic concentration appeared in the case of 1% extract, with cell survival higher than 80% for both cell lines. Interestingly, the impurity profile of polyaniline gel was completely different compared to standard polyaniline powder.

The fact that polyaniline cryogel does not express significant cytotoxicity or embryotoxicity, that various cell types are able to adhere and grow on its surface, and that it can undergo simple surface modification in order to improve its biointerfacial cytocompatibility opens the door to its potential application in regenerative medicine and biosensing.

### Concluding remark

Electrical conductivity, based on the presence of conducting polymer, is an important parameter of cryogels. Though not reported or discussed in the present study, preliminary results suggest that the conductivity of native polyaniline/poly(vinyl alcohol) cryogel swollen with water is of the order of 10^−3^ S cm^−1^ ^12^. In the solutions of electrolytes, especially of acids, such conductivity will be higher due to the contribution of ionic charge-transport. In contrast, polyaniline becomes non-conducting under alkaline conditions when the salt converts to a base, and the contribution of electronic conductivity becomes negligible.

## Conclusions

Polyaniline cryogels supported by poly(vinyl alcohol) are novel macroporous soft conducting materials. They not only have good mechanical integrity represented by Young modulus of 9.7 ± 0.5 kPa but they are also macroporous and highly hydrophilic. All these properties are prerequisites for any application in tissue engineering or biosensing. On the basis of the results of cytotoxicity testing and stem cell differentiation, it can be concluded that polyaniline cryogel also has appropriate biological properties and is therefore suitable for application in tissue engineering and biomedicine in general, where the electrical monitoring or stimulation of tissue is required.

## Electronic supplementary material

Supplementary Information
